# Evaluation of a six-minute walk test in the DE50-MD canine model of Duchenne muscular dystrophy and its effect on blood-borne biomarkers

**DOI:** 10.12688/wellcomeopenres.23269.2

**Published:** 2025-03-17

**Authors:** Dominique Riddell, Rachel Harron, John Hildyard, Dominic Wells, Richard Piercy

**Affiliations:** 1 Comparative Neuromuscular Diseases Laboratory, Clinical Sciences and Services, Royal College Street, London, NW1 0TU, UK; 2 Comparative Biomedical Sciences, The Royal Veterinary College, Royal College Street, London, NW1 0TU, UK

**Keywords:** 6MWT, DE50-MD, DMD, dog model, exercise, biomarkers, serum, Duchenne

## Abstract

**Background:**

Duchenne muscular dystrophy (DMD) is a fatal muscle wasting disease caused by mutations in the dystrophin gene resulting in cycles of muscle degeneration, inflammation and regeneration. The 6-minute walk test (6MWT) is a key functional outcome measure for DMD patient clinical trials and has been adapted for use in animal models of the disease. The DE50-MD dog model of DMD closely reflects the DMD patient phenotype prior to loss of ambulation. For pre-clinical trials using this model, functional outcome measures must be established.

**Methods:**

This longitudinal study compared distance walked in a 6MWT by DE50-MD and WT control dogs and assessed the utility of the 6MWT as a functional biomarker. Dogs underwent two 6MWTs conducted approximately 48-hours apart, at 3, 6, 9, 12, 15 and 18 months of age. In addition, we evaluated the stability of selected blood-borne biomarkers in 12-month old DE50-MD and WT dogs 0, 3, 6, 24 and 48 hours following a 6MWT.

**Results:**

DE50-MD dogs exhibited significantly shorter 6-minute walk distance (6MWD) than WT dogs at all timepoints (P<0.05), with no difference in 6MWD between the first and second 6MWT. C-C motif chemokine ligand 2 (CCL2), myomesin-3 (MYOM3) and myostatin (MSTN) were biomarkers of the DE50-MD phenotype that remained unchanged in DE50-MD dogs following the 6MWT, while creatine kinase (CK) activity significantly increased 3-hours following the test in DE50-MD dogs but remained unchanged in WT dogs.

**Conclusions:**

The 6MWT effectively discriminates DE50-MD from WT dogs aged 3-18 months and a single 6MWT is sufficient for future studies. Serum MYOM3, CCL2 and MSTN are good biomarkers of the DE50-MD phenotype that are unaffected by the relatively low level exertion performed in the 6MWT by 12-month-old DE50-MD dogs.

## Plain language summary

Duchenne muscular dystrophy (DMD) is a fatal muscle wasting disease caused by mutations in the dystrophin gene resulting in cycles of muscle damage, inflammation and repair. The 6-minute walk test (6MWT) is an assessment that measures the distance that a DMD patient can walk in 6 minutes; as the disease becomes more severe with age, affected boys walk progressively shorter distances as muscle function declines. The 6MWT has also been used in animal models of DMD for pre-clinical trials. One such model is the DE50-MD dog model, which has musculoskeletal features that reflect the symptoms manifested by ambulant human DMD patients. In order to use this animal model to trial treatments, functional testing must first be validated. In this study, we compared the distance walked in a 6MWT by DE50-MD and healthy control dogs aged between 3- and 18-months to assess the test’s potential as a measure of muscle function. In addition, we examined the effect of the exercise test on levels of certain blood-borne disease biomarkers. DE50-MD dogs walked shorter distances than the healthy control dogs at all ages. Several blood-borne biomarkers remained stable following the 6MWT (C-C motif chemokine ligand 2 (CCL2), myomesin-3 (MYOM3) and myostatin (MSTN)), while others (such as creatine kinase (CK) activity) increased in the DE50-MD dogs. We conclude that the distance walked during a 6MWT is suitable as a measure of muscle function for future treatment trials using this canine model.

## Introduction

Duchene muscular dystrophy (DMD) is an X-linked disorder in which absence of dystrophin protein (which normally maintains muscle integrity and function) causes progressive skeletal and cardiac muscle degeneration, and death from cardiac or respiratory failure, usually in the third decade of life ^1– 3^. Patients lose their ability to walk significantly earlier, typically from 6 to 15 years of age ^2, 4^. Promising therapies, for example gene editing, gene therapy and exon skipping, are currently at various stages of development or are licenced ^5^. Whilst restoration of dystrophin expression is often a crucial primary endpoint for these approaches, return or maintenance of muscle function represents the ultimate therapeutic objective and functional metrics are thus a key performance measure in trials.

Canine models of DMD, such as the Golden Retriever Muscular Dystrophy (GRMD) model and the canine X-linked muscular dystrophy model Japan (CXMD _J_) are invaluable for preclinical evaluation of treatments as, unlike the mildly affected *mdx* mouse model, they display functional skeletal muscle deficits that more closely reflect those of affected boys ^1^. The DE50-MD dog is a more recently developed canine model, and in contrast to the GRMD and CXMDJ models, the causative mutation in DE50-MD dogs lies within the human mutation “hotspot” region of the dystrophin gene and is amenable to exon 51 skipping or gene editing ^6^, approaches that are applicable to the largest proportion of DMD patients ^5^. Over several years we have been completing a large scale and multifaceted natural history trial of the DMD phenotype in this model. Our work comprises a broad and comprehensive assessment of the DE50-MD phenotype including, but not limited to, skeletal muscle histology ^7^, skeletal muscle MRI ^8^, evaluation of the brain phenotype ^9, 10^, quantification of multiple blood-borne molecules ^11, 12^, and muscle physiology protocols ^13^, all of which have identified promising biomarkers for future pre-clinical trials. Our work to date shows that DE50-MD dogs exhibit disease progression comparable to that of young DMD patients: from first symptomatic manifestation (at approximately 3 years of age) to before loss of ambulation ^7, 8, 11– 13^.

For a test to be useful as a disease biomarker it should be reliable, accurate, reproducible and should enable detection of biologically important differences between affected and unaffected groups. For evaluation of treatment efficacy (therapeutic biomarkers) metrics with low variance are favoured, as these facilitate statistically valid interpretation of even partial improvements, using cohorts with only modest N numbers. Biomarkers with greater variance are correspondingly less helpful (potentially necessitating expensive, impractical and ethically unjustifiable numbers of animals to demonstrate therapeutic benefit). A key component of our work has accordingly been the evaluation of a varied selection of such biomarkers of the DMD phenotype; biomarkers that robustly assess musculoskeletal function are especially relevant as improvement in muscle performance is a key outcome for regulators and most importantly, for patients.

There are various methods currently used to assess functional performance of DMD patients. The North Star Ambulatory Assessment (NSAA), for example, is a rating scale based on the outcome of a range of functional assessments that test the physical abilities of young ambulant boys with DMD ^14^. NSAA assesses patients in key areas such as standing, walking, climbing stairs, jumping, hopping and running ^15^. Further useful measures of muscle function include manual muscle testing (force measured by a trained professional) ^16^, quantitative muscle testing (force measured by a force transducer) ^17^, timed function tests ^18^ and continuous activity monitoring ^19^. However, many of the functional tests used in DMD patients translate poorly to animal models. In contrast, the 6 minute walk test (6MWT) – simply the distance walked by a patient in 6 minutes - is regarded as a key outcome measure in many clinical trials in ambulant boys ^15^; a modified version of the human 6MWT has been used in GRMD dogs ^20^ and in dogs with other diseases ^21, 22^.

Functional testing itself might however, influence the interpretation of other biomarkers. As dystrophic muscle is susceptible to contraction-induced damage, understanding the effect of recent exercise on certain blood-borne biomarkers is important. Serum creatine kinase (CK) activity is a commonly used biomarker for DMD patients ^23^ in particular in the clinical setting, however it is highly labile having a propensity to change following recent exercise ^24, 25^. The effect of exercise on serum CK activity has been demonstrated in animal DMD models including in the *mdx* mouse ^26^ and in GRMD dogs ^27, 28^. Thus, while being a sensitive diagnostic marker, this factor limits its utility in clinical trials: controlling for the amount and type of exercise between patients is challenging. There is therefore a need for identification of serum biomarkers that are less susceptible to exercise-induced fluctuations. We have previously reported multiple blood-borne biomarkers that can discriminate DE50-MD from WT dogs and proposed several (with low variance between animals) that might be applicable to treatment trials ^11, 12^. The effect of exercise on the circulating levels of these DE50-MD biomarkers, however, is yet to be determined.

The aim of this study was firstly to evaluate the potential of the 6MWT as a functional biomarker of the DE50-MD dog phenotype. Secondly, we aimed to test the stability of promising blood-borne biomarkers in response to recent exercise.

## Methods

### Animal husbandry

All dogs were from a colony kept in a dedicated facility, in the Biological Services Unit (BSU) at the Royal Veterinary College (RVC) ^11^ in large pens, with 24 hours a day access to an outside area. The kennel groups were 2 or 3 adult dogs per kennel, and WT and DE50-MD dogs were housed together depending on temperament. All dogs had access to grassy paddocks (approx. 100m ^2^) for up to 7 hours per day, 7 days a week, in groups of no more than 5. Toys, climbing frames and tunnels were rotated through the paddocks (5 in total) for enrichment and to encourage natural behaviours by adding new sights and smells as part of their daily routine. The access to outdoor runs and a grass paddock exceed the minimum stipulations by the *Animal (Scientific Procedures) Act 1986 A(SP)A Code of Practice*.

Carrier female dogs, derived originally from a Bichon-Frise cross Cavalier King Charles Spaniel carrier, were mated with male Beagles (RCC strain, Marshall BioResources) to produce colony dogs; animals used in this study were in their 3 ^rd^ to 4 ^th^ generation. All females whelped naturally and were kept in dedicated whelping rooms at an ambient temperature of 15–24 °C, with a heat lamp (~28 °C) above the whelping box.

At approximately 7 days of age puppies were microchipped; genotype was confirmed by polymerase chain reaction (PCR) performed on a Verity 96 Well Thermal Cyclier (Applied Biosystems) and Sanger sequencing of PCR poducts (SupremeRun, Eurofins Scientific) as previously described ^5^. DNA was derived from buccal swabs and prepared using GeneJET genomic DNA kit (Thermofisher, #K0721). Primers (Eurofins Scientific) were designed spanning the DE50-MD mutation site: Forward primer 5’-3’ sequence AGCTCTGATTGGAAGGTGGT; Reverse primer 5’-3’ sequence ACCTCAGTGTTGTGCTTTTGA. Results were corroborated by measurement of serum CK activity at approximately 2 weeks of age, using a Ilab600 (Instrumentation Laboratory) clinical chemistry analyser (results not shown).

**ARRIVE guidelines:** An E10 checklist was completed for this study (see Extended *data*). ARRIVE guidelines were followed at all times during the design and conduct of the study. All procedures involving study animals were conducted according to UK legislation, within project licence (P9A1D1D6E, granted 11 June 2019) assigned under the Animal (Scientific Procedures) Act 1986. The procedures were also approved by the Royal Veterinary College internal Animal Welfare Ethical Review Body (AWERB). All efforts were made to minimise and ameliorate any animal suffering throughout the study. This was achieved by establishing pre-determined end-points for DE50-MD dogs including dehydration (that could not be resolved by fluid treatment), lethargy, motor dysfunction, weight loss, dysphagia, dyspnoea, listless behaviour or demeanour, or heart failure. Dogs underwent daily observation by animal technician staff. If a dog showed any of these signs they were reported to and assessed by the Study Director, the Named Veterinary Surgeon (NVS) and the Named Animal Care and Welfare Officer (NACWO). The study period ran for 18-months: if a study animal reached any of the pre-determined end-points prior to the planned study end-point, they were humanely euthanised. Euthanasia was performed using an overdose of sodium pentobarbital (250 mg/kg, Dolethal, Covetrus) administered intravenously via a preplaced catheter.

### Six-minute walk test (6MWT)

**Figure 1.**
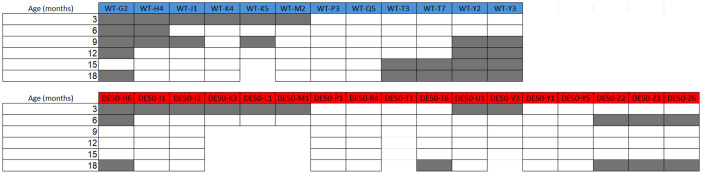
6MWT study population. Age (months) at time of 6MWT for each dog in the study population. Dog ID abbreviations indicate the genotype and the individual dog code: abbreviations beginning with WT are wild-type dogs and those beginning with DE50 are dystrophic DE50-MD dogs. 5 DE50-MD dogs reached humane end points before the end of the 18-month study period (DE50-K3, -L1, -M1, -T1 and -V2) and 1 WT dog (WT-K5) was euthanised at 14-months of age due to reasons unrelated to the study. Boxes shaded in grey indicate that data were not collected at this timepoint.

Male DE50-MD dogs and littermate wild type (WT) control dogs underwent a 6MWT every 3 months up to 18 months of age, starting at 3 months old, each on 2 occasions with the second test occurring between 48 and 72 hours of the first. Data from a total of 29 dogs were used in this study ( Figure 1); 12 WT dogs and 17 DE50-MD. Results were unavailable for some dogs at certain timepoints ( Figure 1) either due to scheduling conflicts, technical issues with data collection, or early animal euthanasia. Five of the 17 DE50-MD dogs were euthanised prior to the end of the 18-month study period due to reaching humane end-points related to dysphagia. One of the 12 WT dogs was euthanised at 14-months of age due to developing steroid-responsive meningitis, as reported previously ^11^, a condition known to affect the Beagle breed ^29^ and believed unrelated to the research. No other humane end-points were reached in any animal.

The 6MWT protocol was adapted from those previously established for DMD patients and the GRMD dog model ^27, 30^. In order to control for differences in limb length (and therefore stride length) between dogs, ulna length was measured (olecranon to the styloid process) prior to each test. Testing was conducted on a flat indoor surface with enough area for the handler and dog to walk comfortably in a 14m loop around cones. Before each test the course was disinfected (Anigene Professional Surface Disinfectant Cleaner ® at a dilution of 1:10 in water) to minimise variation in olfactory cues between animals. Each animal was walked on the handler’s right side, controlled on a leash at the dog’s natural pace around the circuit. Each dog was gently encouraged with voice commands, treats and gentle leash tugs when required but allowed to slow or stop as necessary. The total distance walked in 6 minutes (6MWD) was recorded. Time spent not walking (i.e. standing, sitting or lying down) was recorded throughout the test (as time continued to elapse on the stopwatch) and these periods were combined to give total time not walking during the 6-minute test period.

### Effect of the 6-minute walk test on DE50-MD blood-borne biomarkers

**Figure 2.**
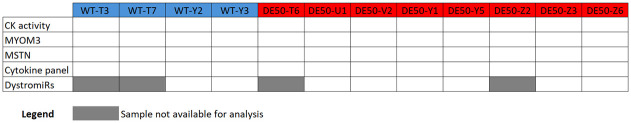
Blood samples tested and dog IDs. Samples analysed for each of the assays performed on blood samples collected before and after a 6MWT in 12 month old dogs. Dog ID abbreviations indicate the genotype and the individual dog code: abbreviations beginning with WT are wild-type dogs and those beginning with DE50 are dystrophic DE50-MD dogs. Analytes measured were CK activity in plasma, and myomesin 3 (MYOM3), myostatin (MSTN), Luminex cytokine/chemokine panel, and dystromiRs (miR-1, miR-133a and miR-206) in serum. Boxes shaded in grey represent samples not available for analysis.

To examine the effect of exercise on blood-borne biomarkers, blood samples were taken before (0 hours) and at 3, 6, 24 and 48 hours following a single 6MWT in 12-month old DE50-MD (n=8) and WT dogs (n=4) ( Figure 2). Blood samples were centrifuged at 500 x g for 10 minutes at 4°C. Serum or plasma (as appropriate) was aliquoted and frozen at -80°C until analysed.

### CK activity

CK activity was quantified in lithium heparinised plasma, using a Ilab600 (Instrumentation Laboratory) clinical chemistry analyser.

### MYOM3 western blot

Western blot was performed as previously described ^11^. Serum samples were diluted 1:1000, and 5μl of diluted sample was used for western blotting. Samples were loaded into Tris/Glycine PAGE gels (7.5% Mini-PROTEAN TGX precast gels, Biorad, #4561026). All samples were tested in duplicate, on replicate gels. Proteins were transferred from the gel to a polyvinylidene difluoride blotting membrane (GE Healthcare, #10600023). Membranes were incubated with a polyclonal rabbit anti-human MYOM3 antibody (Proteintech, #17692-1-AP, 1:1000 dilution) and a polyclonal rabbit anti-canine albumin antibody (as internal loading control, Biorbyt Ltd, #orb242465; 1:1,000,000 dilution) overnight at 4°C. The following day, membranes were washed and incubated with a horseradish peroxidase-conjugated polyclonal goat anti-rabbit IgG secondary antibody (Dako, #P0448, 1:10,000 dilution, 1 hour at room temperature). Membranes were developed using Enhanced chemiluminescence (ECL - ThermoFisher, #32106), and were imaged on a ChemiDoc ^TM^MP Imaging System (Bio-Rad). After imaging, signals were quantified via densitometry using the Fiji distribution of ImageJ software. Probing for MYOM3 gives 2 clear bands at approximately 100 kDa and 140 kDa (as seen in previous studies ^31^, while albumin is detected at approximately 55 kDa. Densitometry data for both MYOM3 bands was combined and normalised to albumin.

### Myostatin ELISA

Myostatin (MSTN) was quantified in serum samples by sandwich enzyme-linked immunosorbent assay (ELISA) according to the manufacturer’s instructions (R&D Systems, #DGDF80).

### Cytokine/chemokine panel

A panel of 13 inflammatory proteins was assayed in multiplex by Luminex assay (Canine Cytokine/Chemokine 13-Plex Array (CD13), performed commercially by Eve Technologies, Canada). These included: granulocyte macrophage colony stimulating factor (GM-CSF); interferon-γ (IFNγ); interleukins (IL) IL-2, IL-6, IL-7, IL-8/CXCL8, IL-10, IL-15 and IL-18; interferon γ-induced protein 10 (IP-10); keratinocyte chemotactic-like (KC-LIKE); C-C motif chemokine ligand 2, also known as macrophage colony stimulating factor 1 (CCL2/MCP-1); and tumour necrosis factor-α (TNFα).

### DystromiR RT-qPCR

microRNAs (miRs) were isolated from serum samples (100–200μl per sample point) using the mRNeasy serum/plasma kit (Qiagen, #217184). cDNA was prepared with the miScript II RT kit (Qiagen, #218161) using 3μl of serum RNA (low RNA content of serum prohibits accurate quantification via nanodrop). All cDNA preparations were then diluted 1/20 with nuclease free water (Fisher Scientific, #10289104).

Following cDNA synthesis, qPCR was performed using the miScript PCR System (Qiagen, #218073) in 10μl volumes (2ul cDNA) using a CFX384 light-cycler (BioRad), with primers specific to *Canis familiaris* dystromiRs miR-1 (Qiagen, #MS00029337), miR-133a (#MS00029498) and miR-206 (#MS00030009) alongside a universal primer (Qiagen, #218073). A melt curve was included in each run. Previous work has shown that miR-223 is a suitable reference gene for comparison of DE50-MD and WT controls aged between 0 and 18 months of age ^11^. As a further QC step, any samples with miR-223 Cq values nearing the stochastic range (Cq>29, indicating low RNA recovery or poor cDNA synthesis) were excluded from analysis: 1 sample (out of 47) was rejected in this manner.

### Statistics

Comparison of 6MWT 1 and 6MWT 2 distances was performed using a two tailed, paired t-test. The correlation between distance walked in 6MWT 1 and 6MWT 2 was assessed by linear regression, accounting for repeated measures. Linear mixed models were used to determine the effects of genotype and age on both absolute and normalised distance walked. Linear mixed models were also used to assess the effect of genotype and time-post 6MWT for CK activity, MYOM3, MSTN, inflammatory cytokines and miRs. Mann-Whitney tests were used to compare pre- and 3-hours post 6MWT results for CK activity and KC-LIKE. The relationships between CK activity and dystromiRs, MYOM3 and MSTN were assessed by linear regression, accounting for repeated measures. For all linear mixed models and linear regression analyses, dog ID was included as a random effect. Post-hoc comparisons were performed using Tukey’s multiple comparisons test. P values of <0.05 were considered significant. Linear regression, linear mixed modelling and *post hoc* analyses were conducted using IBM SPSS Statistics Version 28. T-tests and Mann-Whitney tests were performed and graphs generated using Graphpad Prism 8.0. Free software alternatives could also be used, such as R or JASP. Power/sample size calculations for use of the 6MWD in future trials were performed using GLIMMPSE free online software ^32^, based on a two-way repeated measures ANOVA model comparing the means for each genotype at each timepoint, and using a target power of 0.8 and a type 1 error rate of 0.05.

## Results

### Comparison of 6MWT 1 and 2 distance walked

**Figure 3.**
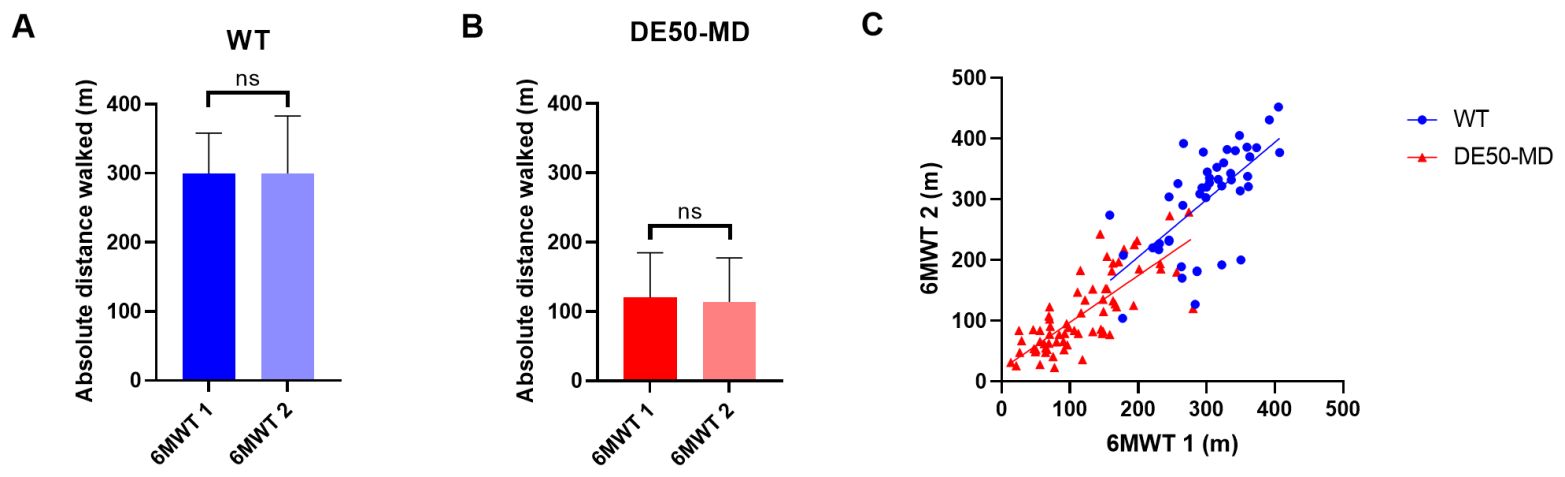
Comparison of results for 6MWTs 1 and 2. Absolute distance walked (mean and standard deviation) during 6MWTs 1 and 2 for **A**) WT dogs (blue; N=12 dogs; N=42 sets of 6MWTs in total; and **B**) DE50-MD dogs (red; N=17 dogs; N=65 sets of 6MWTs in total), and C) the correlation between the results for the 2 tests. No significant difference (ns) in distance walked was observed between the 2 tests in either genotype (P>0.05).

There was no significant difference in the 6MWD between 6MWT 1 and 6MWT 2 across all ages for WT (P>0.3; Figure 3A) or DE50-MD (P>0.4; Figure 3B) dogs; further, a significant correlation between the 2 tests existed for each genotype (P<0.0001; WT: slope estimate: 0.9 +/- 0.1 SE; DE50-MD slope estimate: 0.8 +/- 0.1 SE; Figure 3C). Consequently, further analysis was performed using the mean of the two 6MWDs for each animal.

### 6MWT mean distance walked

**Figure 4.**
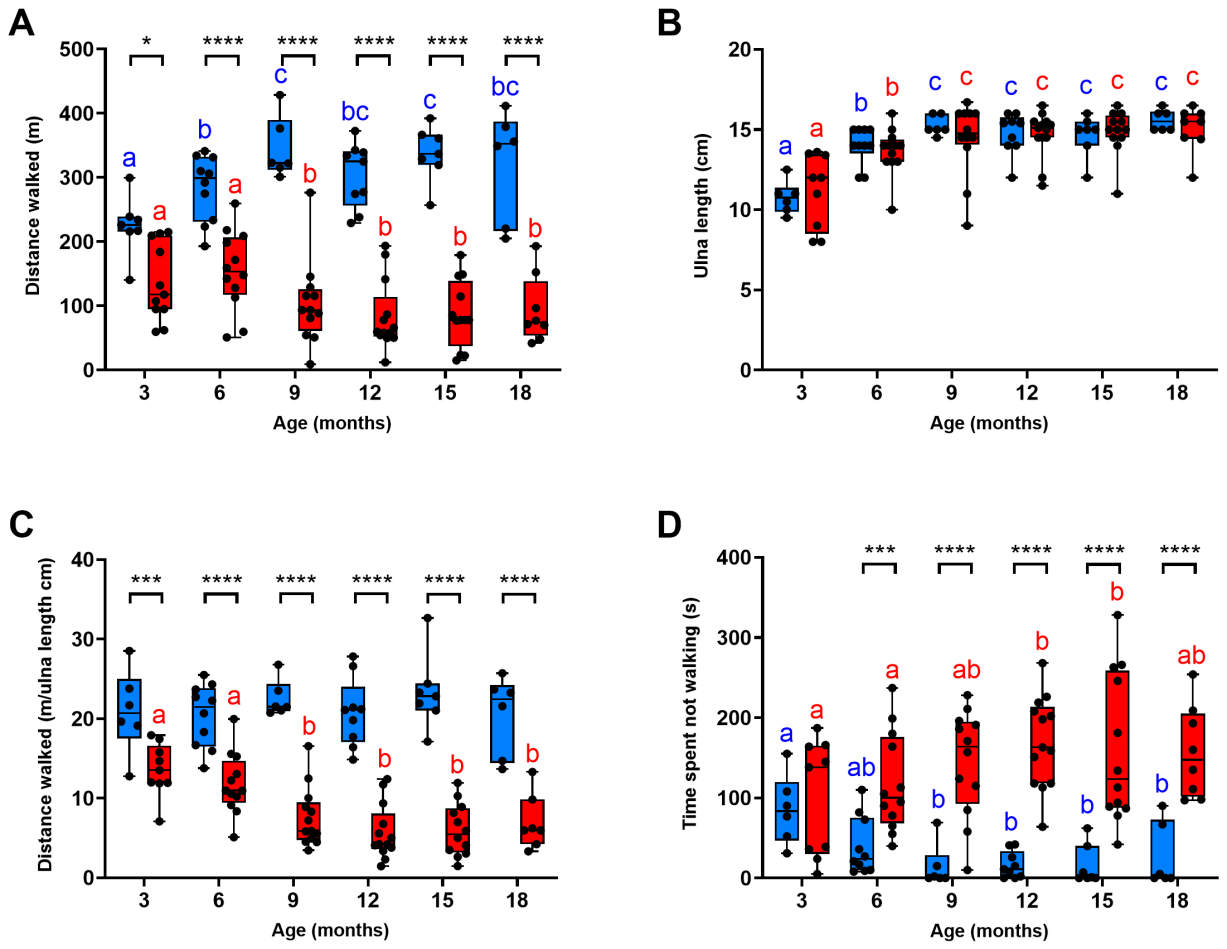
6MWD for DE50-MD and WT dogs. WT (blue) and DE50-MD dog (red) results for **A**) the mean absolute 6MWD for 6MWTs 1 and 2, **B**) ulna length (cm), **C**) 6MWD normalised to ulna length (m/cm), and **D**) mean time spent not walking in seconds (s) for 6MWTs 1 and 2. Boxes extend from the 25 ^th^ to 75 ^th^ percentile, with a line within the box at the median value. DE50-MD: N=17 dogs in total, N=8–13 per timepoint; WT: N=12 dogs in total, N=6–10 per timepoint. Each point represents an individual DE50-MD or WT dog, and whiskers show the minimum and maximum results for that age-group. Red letters (a and b) denote statistically significant differences (P<0.05) in the mean within the DE50-MD genotype and blue letters (a, b and c) represent statistically significant differences (P<0.05) in the mean within the WT genotype: means sharing a letter are not significantly different. Asterisks denote the level of significance of a difference between genotypes based on linear mixed model analysis, adjusted for repeated measures: *P<0.05, ***P<0.001, ****P<0.0001.

The absolute mean distance walked (mean of walk test 1 and walk test 2) was reduced in DE50-MD compared to WT dogs at all ages (P<0.0001; Figure 4A). The difference between genotypes became more apparent with age. 6MWD for WT dogs increased progressively between 3 and 9 months of age, at which point it plateaued through to 18 months. In contrast, the greatest distances walked by the DE50-MD dogs were achieved at 3 to 6 months, after which the 6MWD decreased and plateaued from 9 to 18 months.

Given that 6MWD is influenced strongly by stride length, we normalised distances to ulna length. Lengths increased from 3 to 9 months in all animals (reflecting growth and maturation) but were not significantly different between genotypes at any age (P=0.88; Figure 4B). The mean 6MWD normalised to ulna length (cm) was remarkably consistent with age in WT dogs, suggesting that essentially all age-associated increases in walk distance were attributable to growth. Notably, distance walked remained significantly greater than that of DE50-MD dogs at all ages (P<0.0001; Figure 4C), and indeed normalised DE50-MD data exhibited the same essential pattern as raw data, with dystrophic dogs walking greater distances at 3–6 months than at later ages. Conversely, time spent not walking during the test was greater for DE50-MD compared to WT dogs (P<0.0001) at all timepoints except 3-months ( Figure 4D). WT dogs spent the longest periods not walking at 3 and 6 months of age, after which they were stationary for relatively little time. In contrast, DE50-MD dogs’ still episodes were of similar total duration to WT dogs at 3 months, after which the genotypes diverged with DE50-MD dogs spending the greatest period not walking at older ages (9-months onwards).

Sample size calculations showed that for non-normalised and ulna-length normalised 6MWD a sample size of 6 dogs per genotype would detect with high power (>0.8) a 50% change in DE50-MD 6MWD towards that of WT dog 6WMD ( Table 1) in possible future treatment trials. 10 or more animals would be required to detect an improvement of 25% in 6MWD in DE50-MD dogs.

**Table 1.** 6-minute walk distance (6MWD) power calculations. Group size denotes the number of dogs required per genotype (WT and DE50-MD) and associated power to detect a significant change in 6MWD with any future treatment. Percentage change detected (%) for a 100%, 75%, 50% or 25% change in DE50-MD results towards those of WT results for 6MWD (m) and 6MWD normalised to ulna-length (m/cm).

Outcome	Parameter	Percentage change detected (%)			
		100%	75%	50%	25%
6MWD (m)	Group size	4	4	6	10
	Power	0.97	0.86	0.96	0.83
6MWD (m/cm)	Group size	4	4	6	12
	Power	0.96	0.84	0.95	0.88

### Effect of the 6-minute walk test on DE50-MD blood-borne biomarkers

For the 6MWT blood biomarker response component of the study, samples were collected from 12-month-old animals. At this age, DE50-MD dogs (N=8) walked a significantly shorter distance than WT dogs (N=4), as expected (P<0.0001); WT dogs achieved a mean 6MWD of 337m +/- 20.55 SD (range: 308–353m), while the DE50-MD group achieved a mean 6MWD of 93m +/- 38 SD, (range: 47–154m) ( Table 2, Figure S1). Three out of the 4 WT dogs walked for the full 6-minute duration of the test, (the fourth WT dog (WT-Y3) spent 50 seconds stationary in total). In contrast, all DE50-MD dogs (N=8) spent a proportion of the test not walking (range 120–276 seconds spent either standing still, sitting or lying down). Thus, as expected, the total amount of exercise performed by each genotype group differed.

**Table 2.** Summary of data collected from the 6MWT blood biomarker response study. WT: wild-type; m: metres; s: seconds; cm: centimetres.

Dog ID	Genotype	Distance Walked (m)	Time not walking (s)	Length of Ulna (cm)	Distance walked/length of ulna (m/cm)
WT-T3	WT	336	0	15.0	22.40
WT-T7	WT	353	0	15.5	22.77
WT-Y2	WT	308	50	15.0	20.53
WT-Y3	WT	350	0	15.0	23.33
DE50-T6	DE50-MD	77	210	14.0	5.50
DE50-U1	DE50-MD	154	172	14.5	10.62
DE50-V2	DE50-MD	70	262	15.5	4.52
DE50-Y1	DE50-MD	47	276	15.0	3.13
DE50-Y5	DE50-MD	144	120	16.5	8.73
DE50-Z2	DE50-MD	80	220	16.0	5.00
DE50-Z3	DE50-MD	70	221	16.5	4.24
DE50-Z6	DE50-MD	98	217	16.0	6.13

**Figure 5.**
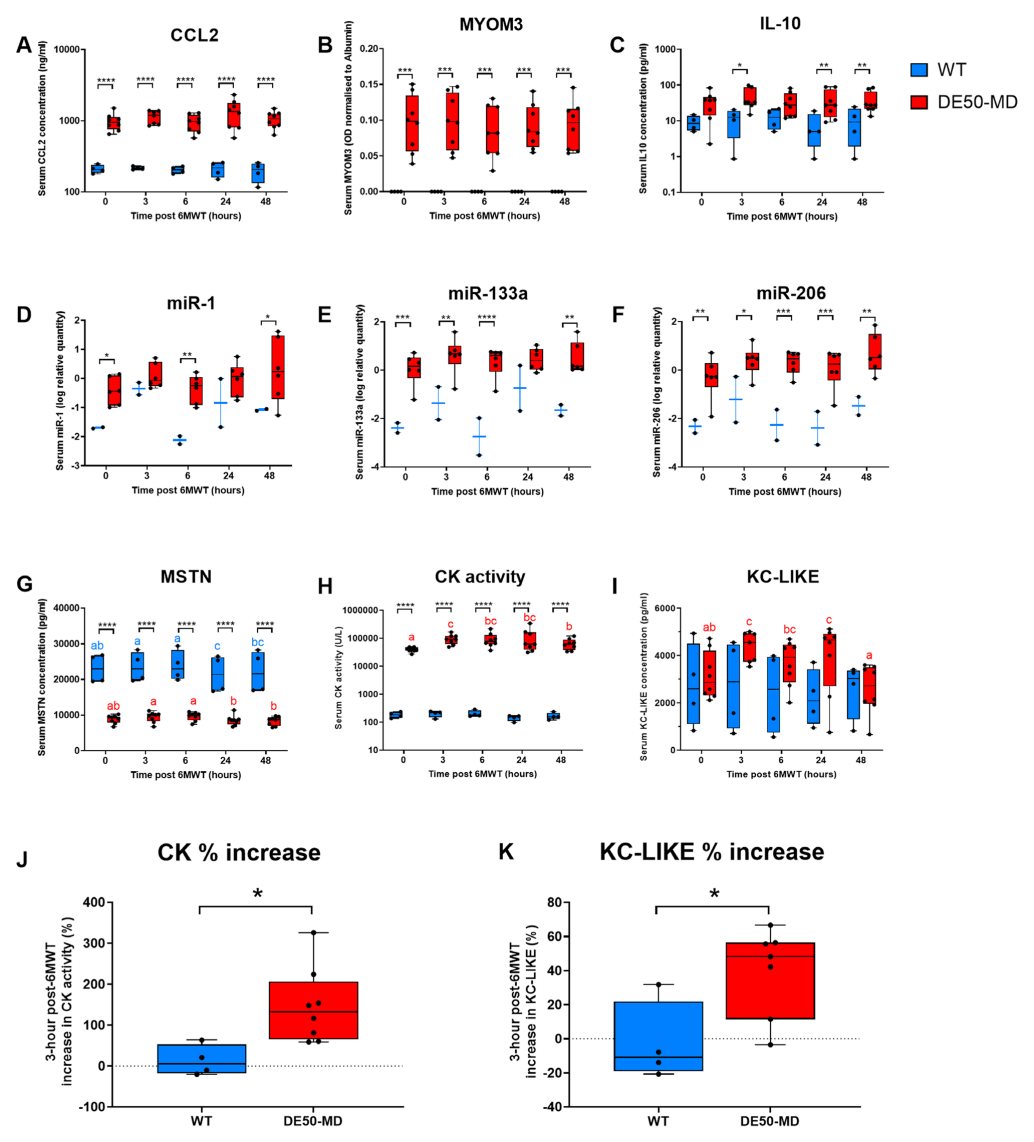
Blood-borne biomarkers before and after the 6MWT in DE50-MD and WT dogs. Results for analytes of interest for WT (blue) and DE50-MD dogs (red). **A**) CCL2 Luminex assay results (ng/ml), **B**) MYOM3 western blot results normalised to albumin (AU), **C**) IL-10 Luminex assay results (ng/ml), **D**) miR-1 RT-qPCR log relative quantity, **E**) miR-133a RT-qPCR log relative quantity, **F**) miR-206 RT-qPCR log relative quantity, **G**) MSTN ELISA results (pg/ml), **H**) CK activity (Units/L), **I**) KC-LIKE Luminex assay results (ng/ml), **J**) percentage change in CK activity 3-hours post-6MWT, **K**) percentage change in KC-LIKE 3-hours post-6MWT. DE50-MD N=8, WT N=4 for graphs **A**– **C** and **G**– **K**; DE50-MD N=6, WT N=2 for graphs **D**– **F**. Boxes extend from the 25 ^th^ to 75 ^th^ percentile, with a line within the box at the median value. Each point represents an individual DE50-MD or WT dog, and whiskers show the minimum and maximum results for that age-group. Letters a, b and c denote statistically significant differences (P<0.05) in the mean within the DE50-MD (red letters) or WT (blue letters) genotype: means sharing a letter are not significantly different. Asterisks denote the level of significance of a difference between genotypes based on linear mixed model analysis, adjusted for repeated measures: * P<0.05, **P<0.01, ***P<0.001, ****P<0.0001.

Biomarkers quantified included the skeletal muscle proteins CK, MYOM3 and MSTN, the dystromiRs miR-1, miR-133a and miR-206, and the inflammatory proteins CCL2, KC-LIKE, GM-CSF, IFNγ, ILs-2, -6, -7, -8, -10, -15, -18, TNFα and IP-10. Of these, CCL2 (P<0.0001), MYOM3 (P=0.0004), IL-10 (P=0.009), miR-1 (P<0.0001), miR-133a (P=0.0003), miR-206 (P<0.0001) and CK activity (P<0.0001) were significantly higher overall in DE50-MD compared to WT blood, while MSTN was significantly lower in DE50-MD dogs compared to WT (P<0.0001) ( Figure 5 and Figure S2). Quantified proteins that did not significantly differ overall between the two genotypes were KC-LIKE (P=0.18), GM-CSF (P=0.14), IFNγ (P=0.54), IL-2 (P=0.22), IL-6 (P=0.25), IL-7 (P=0.26), IL-8 (P=0.31), IL-15 (P=0.13), IL-18 (P=0.30) and TNFα (P=0.42). IP-10 was below the lower-limit of quantification for all samples tested (<3 ng/ml) (Figures S3 and S4).

Following completion of the 6MWT, 6 biomarkers remained stable and unchanged from baseline in both genotypes over 48-hours: CCL2, MYOM3, IL-10, miR-1, miR-133a and miR-206. Both CCL2 ( Figure 5A) and MYOM3 ( Figure 5B) were markedly increased in DE50-MD compared to WT dogs pre-6MWT (0-hours) and remained stable throughout the sampling period in both genotypes. Similarly, for both WT and DE50-MD genotypes IL-10 levels did not fluctuate significantly over 48-hours. In contrast to CCL2 and MYOM3 however, while IL-10 was elevated in the DE50-MD population overall, here the difference between genotypes did not reach significance at all time points (including at baseline).

Like CCL2 and MYOM3, all 3 dystromiRs were markedly elevated in DE50-MD dogs before commencement of the 6MWT (0-hours) ( Figure 5 D–F). The dystromiRs showed some mild variation in serum quantity over the 48-hour period, with mean serum concentrations of all 3 dystromiRs increasing 3 hours post 6MWT in DE50-MD dogs, although this increase was not statistically significant. Due to a low sample size for dystromiR quantification for WT dogs (N=2), statistical comparisons within the WT group were not performed. However, all 3 dystromiRs shared a similar pattern with time across both genotypes.

Of all the measured analytes, MSTN was the only biomarker that was lower in DE50-MD compared than in WT dog samples ( Figure 5G) at baseline and throughout the 48-hour sampling period. MSTN did fluctuate modestly over the 48-hour period in both groups (P<0.05), however the effect of time did not significantly differ between genotypes (P=0.41).

Both CK activity (P=0.03) and KC-LIKE (P=0.04) were the only 2 biomarkers to significantly increase in DE50-MD dogs following the 6MWT, while remaining unchanged at all timepoints in WT dogs ( Figure 5H–I). In DE50-MD dogs, CK activity increased 3-hours post-6MWT from a mean of 40,000 U/L (+/-7000) to 96,000 U/L (+/-37,500) ( Figure 5H), with a mean percentage increase of 145% (range: 58–326%) in DE50-MD dogs and only 13% (range: -20–63%) for WT dogs ( Figure 5J). CK activity remained significantly elevated above pre-6MWT levels throughout the 48-hour study period in DE50-MD dogs ( Figure 5H). KC-LIKE protein was the only protein in the cytokine panel that increased significantly in DE50-MD dogs following the 6MWT, with a mean percentage increase of 40% (range: -3–67%) in DE50-MD dogs and -3% (range: -21-32%) in WT dogs between 0- and 3-hours post-6MWT ( Figure 5I and Figure 5K). Despite this increase, KC-LIKE concentration was still comparatively variable overall (in both genotypes) and was consequently not significantly different between healthy and dystrophic animals at any timepoint. KC-LIKE concentrations moreover returned to baseline levels in the DE50-MD group by 48-hours post-6MWT.

**Figure 6.**
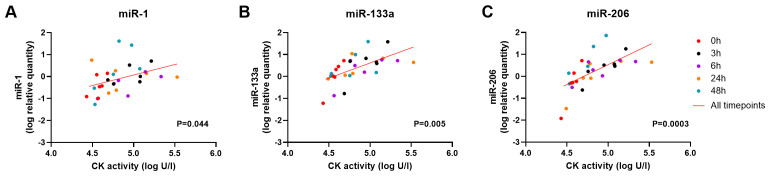
Relationship between CK activity and dystromiRs in the blood of DE50-MD dogs that have performed a 6MWT. Linear regression analysis of plasma CK activity (log units/l) with serum dystromiRs **A**) miR-1, **B**) miR-133a and **C**) miR-206 (log relative quantity), in dogs that have performed a 6MWT. Data from blood collected from N=6 DE50-MD dogs at 0h (red), 3h (black), 6h (purple), 24h (orange) and 48h (green) following completion of a 6MWT. P value for the linear regression analysis of all timepoints combined, accounting for repeated measures (red line).

Linear regression analysis of CK activity with dystromiRs -1, -133a and -206 showed that a significant positive relationship was present with miR-1 (P= 0.044, slope gradient estimate: 0.95 +/- 0.5 SD), miR-133a (P=0.005, slope gradient estimate: 1.3 +/- 0.4 SD ) and miR-206 (P=0.0003, slope gradient estimate: 1.8 +/- 0.5 SD) in DE50-MD dogs that underwent a 6MWT ( Figure 6). No relationship was found between CK activity and muscle proteins MYOM3 or MSTN (P=0.97 and P=0.24 respectively, Figure S5).

## Discussion

Tests that quantitate muscle function in animal models are key components for the translation of potential DMD treatments; whilst major changes in mortality and longevity are long desired outcomes in DMD, even modest improvements in muscle performance could offer significant changes to quality of life. The ability to detect even relatively small differences in musculoskeletal function reflects a critical advantage of canine DMD models when compared with rodent models when assessing a treatment’s potential efficacy. Accordingly, we sought to validate use of the 6MWT as an objective biomarker of muscle function in the DE50-MD model. Further, the study design allowed us to evaluate the effect of the 6MWT on selected blood-derived biomarkers, aiding interpretation of biomarker data in a post-exercise context.

We first sought to assess the short-term variability of results of the 6MWT by conducting the test on two separate occasions, with the second test occurring between 48–72 hours after the first. Repeatability of the 6MWT has been demonstrated in DMD boys aged 5–12 years, with a very strong correlation for distance walked in two tests carried out between one and three weeks apart ^30, 33^. Indeed, a multicentre DMD patient clinical trial showed that of the muscle function tests used (6MWT, timed function tests and myometry) the 6MWT had the best test-to-test reproducibility ^18^. As reported in DMD patients, both WT and DE50-MD dogs had a strong positive correlation between absolute distance walked during the first and second 6MWT, regardless of age. Based on the reproducibility of these results, we conclude that a single 6MWT could be considered representative of walk test performance for any given age (3–18 months).

When comparing DE50-MD and WT genotypes, DE50-MD dogs walked significantly shorter absolute and leg length-adjusted distances at all ages tested. Dogs from the DE50-MD colony reach their adult height at approximately 9-months of age; ulna length increased between 3 and 9 months before plateauing and was used as a proxy for stride length in this study. Stride length is a key determinant of 6MWD in both healthy boys and DMD patients ^30^ and accordingly, when WT dog 6MWD was normalised to ulna-length it eliminated any age-associated changes. Within the DE50-MD group however, though ulna-length adjustment reduced variation between subjects, it did not significantly alter the age-associated decline seen for absolute distance walked. This reveals the functional decline that occurs in ambulatory muscles as the DE50-MD dogs get older.

Prior to starting the study, the effect of dog age on compliance with the test protocol was a concern. In healthy children, age influences 6MWT compliance, with children aged between 3 to 5 years having less than a 39% test completion rate, compared to greater than 93% for children over the age of 6 ^34^. The earliest timepoint in our study was at 3-months of age: an age at which the puppies were still developing, were easily distracted and had had relatively little lead-training. In WT dogs, time spent not walking was greatest at younger ages (3 and 6 months), compared to older animals (9 months onwards) suggestive of an element of developmental maturity and perhaps learnt experience of the 6MWT. This effect was not seen in DE50-MD dogs, where time spent stationary typically increased with age, suggesting that contributions from disease progression were sufficient to render training-associated differences irrelevant. Despite these contrasting contributions, differences in 6MWD between genotypes were evident even in dogs as young as 3 months. The marked differences between genotypes at all ages suggest that the 6MWT is an excellent biomarker of the DE50-MD phenotype. Further, the relatively small sample sizes required to detect up to a 50% improvement in 6MWD demonstrates a strong potential for the 6MWT as a primary outcome measure of muscle function in future pre-clinical studies in this dog model.

There were some limitations of our study. Importantly, the test walkers could not be blinded to genotype, as dystrophic animals are readily discerned via their physical characteristics and relative degrees of muscle atrophy ^7, 8^. Consequently, the handlers might have been less likely to encourage the DE50-MD dogs to continue walking (both dog groups typically required vocal encouragement to stay engaged with the task). Secondly, dogs were walked on a leash by a handler: it is conceivable that the handler might have influenced the test in other ways, such as through variation in their own stride length or through other actions. Nevertheless, the large number of dogs recruited and the longitudinal structure of this study, together with the marked difference between genotypes and limited variation within genotypes (despite the caveats discussed above), gives confidence in the robustness of this biomarker to differentiate between DE50-MD and WT dogs. A potential confounding factor is the cognitive phenotype of the DE50-MD dog model: we have previously shown that, while DE50-MD dogs demonstrate comparable rates of learning new tasks to WT dogs, they show reduced focus on the tasks ^9^. DE50-MD dogs also display reduced exploratory behaviour compared to age-matched WT dogs. These factors have the potential to influence the 6MWT: time spent not walking could be due to muscle functional deficits or a loss of focus or interest in the test, or both. Indeed, DMD patients with concomitant neurodevelopmental disorders such as autism or attention-deficit/hyperactivity disorder achieve significantly lower motor function test scores than those without ^35^. For future studies, use of objective data derived from activity monitoring could avoid potential handler bias, as well as eliminating the need to perform a specific task that may be influenced by a cognitive phenotype ^36^. Such measurements can be conducted over longer periods and in the dogs’ natural environment, and without the need for direct human interaction.

This study allowed us to assess the effect of recent exercise on selected blood-borne biomarkers that we consider important for future pre-clinical translational trials ^11, 12^. Although the duration of each test was the same between groups, the total amount of exercise differed between groups, meaning that within group effects are likely most relevant. Indeed, exercise resulted in a significant increase in serum CK activity following the 6MWT in the DE50-MD dogs but not in the WT group, even though the total distance walked by the dystrophic animals was lower than that of WT dogs. Previous studies have concluded that the mechanism underlying exercise-induced elevations in CK activity appears to be micro-lesions in the dystrophic sarcolemma, allowing the high-abundance protein to be leaked from muscle into the extracellular space ^26^. Our result demonstrates the high sensitivity of CK activity as a marker of dystrophic muscle damage following even mild exercise in the DE50-MD model, in accordance with findings in *mdx* mice ^25^, the GRMD dog model ^27^ and DMD patients ^37^. Interestingly, a 6MWT study in the GRMD model found a significant increase in serum CK activity immediately after a 6MWT in 6-month-old but not 12-month-old dogs ^27^. It is a limitation of our study that we only measured biomarker-responses to recent exercise in 12-month dogs. As both DE50-MD and GRMD dogs walk greater absolute and stride-length adjusted 6MWDs at 6-months compared to 12-months, it is possible that post-exercise percentage increase in serum CK activity might have been even higher had the test been performed in 6-month old DE50-MD dogs.

For pre-clinical studies evaluating the efficacy of treatments, having biomarkers that are not influenced by exercise eliminates exertion as a confounding factor. One such candidate is MYOM3, a structural protein located at the M-band of striated muscle ^38^. MYOM3 was significantly elevated in the serum of all DE50-MD dogs compared to WT dogs, and levels were unchanged in the 48 hours following the 6MWT. MYOM3 could be used as an alternative to CK activity as a marker of muscle structural damage ^31^. Previous work has shown that compared to CK activity, serum MYOM3 has lower variation between and within DE50-MD dogs ^11^, while the current study suggests that it is also less influenced by physical activity. Whether serum MYOM3 might be affected by a more strenuous exercise bout remains to be determined, however we note that for DE50-MD dogs in particular the 6MWT already represents a substantial increase in focussed exercise over their otherwise more voluntarily sedentary daily activity. As such, MYOM3 remains a useful adjunct biomarker that appears unaffected by recent muscle exertion.

Another biomarker that remained stable following the 6MWT was CCL2, a chemokine that is expressed by inflammatory cells as well as by injured skeletal muscle itself ^39, 40^. CCL2 was the only inflammatory protein quantified that was elevated in DE50-MD dogs compared to WT dogs prior to exercise. While increased transcription of inflammatory proteins has been observed following acute exercise in *mdx* mice ^41^, we observed no increase in CCL2 in the 48 hours following the 6MWT. KC-LIKE protein, another chemokine produced by both leukocytes and damaged muscle ^42^, was modestly increased 3 hours following exercise (1.4-fold), however serum concentrations remained within WT limits at this time-point. Cytokine IL-10 was the only other protein on the cytokine panel that was significantly elevated in DE50-MD compared to WT dogs. Although serum concentrations of IL-10 did not vary significantly pre- or post-6MWT, *post-hoc* analysis showed that baseline levels of IL-10 were not significantly higher than in WT dogs. Previous work evaluating this biomarker showed that while serum IL-10 was elevated when analysing 3–18 month old dogs in a repeated measures’ design, differences between 12-month old dogs at this age alone were not significantly different, minimising its utility as a biomarker in trials using this age-group ^12^. Overall, we conclude that of the inflammatory biomarkers, serum CCL2 is a robust marker of the DE50-MD phenotype that is likely to remain stable under the levels of physical activity conducted here. Whilst more intense exertion might elicit greater muscle damage and associated inflammation, it is unlikely to be of relevance in the context of the DE50-MD model otherwise voluntarily exercising.

MSTN and dystromiRs -1, -133a and -206 are biomarkers in DMD patients and animal models including in the DE50-MD dog model ^11, 43– 45^. MSTN is secreted by skeletal muscle and regulates muscle growth via inhibition ^46^, while dystromiRs are skeletal muscle-specific microRNAs that are elevated in the serum in DMD patients and animals ^43, 44, 47^. At baseline, MSTN concentration was significantly reduced in DE50-MD serum, while all 3 miRs had higher concentrations in DE50-MD compared to WT serum, as expected based on previous studies ^11^. MSTN and the dystromiRs showed mild, non-significant fluctuations over the 48-hours sampling period, and the association between serum quantities of these biomarkers and time post-6MWT did not differ significantly between the two genotypes. Mean serum dystromiR concentrations were nevertheless higher at 3 hours following the 6MWT in DE50-MD dogs, and while these differences were not statistically significant, there was a positive correlation between each of the dystromiRs with CK activity over the 48-hour sampling period (with this latter biomarker showing clear exercise-associated increases). We have previously demonstrated a correlation between each of the 3 dystromiRs and CK activity within blood samples of dogs in our DE50-MD population as part of our natural history study ^11^. The work in our present study suggests that, while both circulating CK activity and dystromiR levels are sensitive markers of the DE50-MD phenotype, their serum content might be influenced by mild exercise over similar timescales. Typically, serum dystromiRs are at very low levels in healthy subjects, however increased serum miR-1, miR-133a and miR-206 following exercise has been demonstrated, with peak change in CK activity correlating with peak change in circulating levels of all 3 miRs in an exercise study of healthy volunteers ^48^. This suggests that miR-1, miR-133a and miR-206 are only released from muscle as a consequence of tissue damage (similar to the mechanism proposed for CK). In contrast, DE50-MD serum MSTN showed no correlation with CK activity. Based on these results, we conclude that in future studies comparisons of DE50-MD and WT dystromiR concentrations could be influenced by mild exercise, while MSTN results are unlikely to be affected.

In summary, the 6MWT reliably discriminated DE50-MD from WT dogs and shows strong potential as a primary functional outcome measure for future pre-clinical trials. Furthermore, our biomarker-response study in 12-month-old dogs showed that serum CCL2, MYOM3 and MSTN concentrations are useful biomarkers of the DE50-MD phenotype that are unaffected (CCL2 and MYOM3) or minimally affected (MSTN) by the low levels of physical activity performed by this age group. In contrast, CK activity was increased in DE50-MD blood samples following even mild activity and correlated with serum dystromiR levels; it might be that muscle repair needs to be considerable (or complete) before the effect of exercise is abrogated with these sensitive biomarkers. Finally, alternative measures of musculoskeletal activity, such as use of collar-mounted accelerometers that can record without requirement for a handler, might help reduce any bias that occurs with the canine 6MWT test and provide useful, adjunctive data.

## Ethics statement

All of the experimental procedures that involved animals in this study were conducted according to UK legislation, within a project licence (P9A1D1D6E, granted 11 June 2019) assigned under the Animal (Scientific Procedures) Act 1986 and approved by the Royal Veterinary College Animal Welfare Ethical Review Body (AWERB).

## Consent statement

Consent were not required

## Data Availability

Figshare: Evaluation of a six-minute walk test in the DE50-MD canine model of Duchenne muscular dystrophy and its effect on blood-borne biomarkers – version 1’ 10.6084/m9.figshare.27135216 ^49^. This project contains the following underlying data: 6MWT – raw full data Blood biomarker – key biomarker summary Blood biomarker – MYOM3 raw data Blood biomarker - MSTN raw data Blood biomarker – microRNA RT-qPCR raw data Figshare: Evaluation of a six-minute walk test in the DE50-MD canine model of Duchenne muscular dystrophy and its effect on blood-borne biomarkers – version 1 10.6084/m9.figshare.27135216 ^49^ This project contains the following extended data 6MWT – supplementary data: figures S1–S5 Blood biomarker – supplementary biomarker summary Arrive guidelines E10 checklist Data are available under the terms of the Creative Commons Attribution 4.0 International license (CC-BY 4.0).

## Software availability statement

- R open access software available from:
  https://www.r-project.org/
- JASP open access software available from:
  https://jasp-stats.org/
- GLIMMPSE open access software available from:
  https://glimmpse.samplesizeshop.org/
- Fiji distribution of ImageJ open access software available from:
  https://imagej.net/software/fiji/downloads
