# Supplementary material for: Evaluation of a six-minute walk test in the DE50-MD canine model of Duchenne muscular dystrophy and its effect on blood-borne biomarkers
Source: Wellcome Open Res. 2025 Mar 17;9:681. Originally published 2024 Nov 14. [Version 2] doi: 10.12688/wellcomeopenres.23269.2 (PMC12022548; doi:10.12688/wellcomeopenres.23269.2)
Supplement: Peer review history

# Reviewer report: Reviewer response for version 2

**DOI:** 10.21956/wellcomeopenres.26410.r121945

**Published:** 2025-04-24

**Authors:** Yuri van der Burgt

**Affiliations:** 1 Leiden University Medical Center, Leiden, The Netherlands

In this manuscript the authors report on the evaluation and applicability of a six-minute walk test in the DE50-MD canine model of Duchenne muscular dystrophy (DMD). The study is well-presented and the report is sound and clear. Although the scientific quality of this work is very much appreciated, it may not be obvious how this study will contribute to a more clinical aim to improve DMD patient care. Clearly the use of an animal model adds to our knowledge of the disease and as the authors write “canine models are invaluable for preclinical evaluation of treatments”. The value of using a canine model is undisputed, but the concerns of this reviewer relate to whether a walk test is suitable for evaluations of the animal model. In the abstract the authors correctly state that  “The 6-minute walk test (6MWT) is a key functional outcome measure for DMD patient clinical trials”, but why a similar walk test the desired way-to-go for evaluation of the canine model? This reviewer agrees with a concern raised by a previous reviewer: “The 6MWT is highly standardized in humans, in particular the instruction to walk as far as possible… It seems difficult to force dogs to maintain a walking gait, and perhaps a bit artificial, so how was this done?”. The authors are encouraged to work on more objective parameters, such as molecular biomarkers. Indeed they include work on such markers, but unfortunately the focus lies on whether or not such biomarkers change as a results of exercise, in this case the walk test itself (…). It is recommended to perform the work on the identification and development of biomarkers that can replace a subjective walk test (that varies day-to-day with ability and motivation). Moreover, it is not clear why we should measure such markers after the walk test (“There is therefore a need for identification of serum biomarkers that are less susceptible to exercise-induced fluctuations.” and “The effect of exercise on the circulating levels of these biomarkers is yet to be determined.”). Instead of evaluating the effect of exercise on the markers these should rather be used to monitor therapeutic interventions and measured in a longitudinal manner. The authors conclude that “The 6MWT effectively discriminates DE50-MD from WT dogs aged 3-18 months and a single 6MWT is sufficient for future studies.”, but such a differentiation is not the purpose of the walk test. Note that 6MWT is meant for disease monitoring rather than diagnostic purposes. This comment also affects the title that should rather read:

 “Evaluation of a six-minute walk test and circulating biomarkers in the DE50-MD canine model of Duchenne muscular dystrophy”

 A less important comment concerns the term “blood-borne biomarker”. Although this reviewer acknowledges not to be a native speaker, the term blood-borne is more common for infectious diseases and moreover misleading. Please just refer to these markers as “blood-based biomarkers” or “circulating biomarkers”.

Is the work clearly and accurately presented and does it cite the current literature?

Yes

If applicable, is the statistical analysis and its interpretation appropriate?

Yes

Are all the source data underlying the results available to ensure full reproducibility?

Yes

Is the study design appropriate and is the work technically sound?

No

Are the conclusions drawn adequately supported by the results?

No

Are sufficient details of methods and analysis provided to allow replication by others?

Yes

Reviewer Expertise:

Biomarker research, analytical chemistry, clinical proteomics

I confirm that I have read this submission and believe that I have an appropriate level of expertise to confirm that it is of an acceptable scientific standard, however I have significant reservations, as outlined above.

# Reviewer report: Reviewer response for version 1

**DOI:** 10.21956/wellcomeopenres.25648.r112850

**Published:** 2024-12-31

**Authors:** Ines Barthélémy

**Affiliations:** 1 Univ Paris-Est Créteil, Créteil, France

The study reported in this manuscript aims to assess the potential interest of adapting the 6 MWT, the gold standard outcome measure in DMD clinical trials, to the DE50-MD canine model of DMD. A secondary objective of the study is to determine whether various blood-borne biomarkers, previously validated in the model, are affected by the 6MWT exercise. The 6-minute walking distance was reduced in affected dogs and the difference compared to WT dogs increased with age. The biomarker study was performed at only one time point, at 12 months of age. A slight increase in CK was observed and the only biomarker that also changed significantly after exercise was the cytokine KC-LIKE, although it was not significantly different from WT dogs. Variation in DystromiRs correlated with CK but was not significant. The authors conclude that the 6MWT is a suitable assessment method for their canine model and that some biomarkers such as MYOM3, CCL2 and MSTN are not influenced by exercise. The influence of occasional exercise to induce fluctuations in biomarkers is an important issue to be addressed before a biomarker is routinely used in DMD patients or animal models, and therefore such a study is of interest. However, I wonder why the authors chose to perform this biomarker study at 12 months, which is quite late in the life of the dogs in terms of their clinical evolution. At an earlier time point, the CK elevation would probably have been more pronounced, and possibly that of other biomarkers, at least the dystromiRs and inflammatory cytokines. Therefore, I think that the conclusion that MYOM3 and CCL2 are not affected by exercise should be drawn with more caution, because it might be different at earlier time points with a more pronounced exercise-induced CK increase.

 -On the 6 MWT:

 How is the time not walking measured?

 Are the tests filmed and is video analysis used?

 The 6MWT is highly standardized in humans, in particular the instruction to walk as far as possible. Has the test been performed in a walking gait in dogs? It seems difficult to force dogs to maintain a walking gait, and perhaps a bit artificial, so how was this done?

 Do the authors think that cognitive impairments, which they have already described in their model, might affect the performance of DE50-MD dogs in this test?

 - About the biomarkers

 The CK elevation seems mild, but this may be due to the graphical presentation. I would suggest adding a Y-axis cut-off in Figure 5H to make it clearer. It would also be useful to add information about the range of elevation in the results section.

 I question your decision to perform this analysis at 12 months of age rather than earlier, when the dogs still have myofibers to break down. The CK increase would probably have been more pronounced and the assessment of the sensitivity of the other biomarkers to exercise would have been more reliable.

 In the paper published by Acosta *et al*. in 2016 on the 6 MWT in GRMD dogs and its effect on CK levels, significant change in CK was detected after the 6 MWT at 6 months of age, but not at 12 months of age. This should be included in the limitations of the study in the Discussion section.

Is the work clearly and accurately presented and does it cite the current literature?

Yes

If applicable, is the statistical analysis and its interpretation appropriate?

Yes

Are all the source data underlying the results available to ensure full reproducibility?

Yes

Is the study design appropriate and is the work technically sound?

Partly

Are the conclusions drawn adequately supported by the results?

Partly

Are sufficient details of methods and analysis provided to allow replication by others?

Partly

Reviewer Expertise:

neuromuscular diseases modelling

I confirm that I have read this submission and believe that I have an appropriate level of expertise to confirm that it is of an acceptable scientific standard, however I have significant reservations, as outlined above.

# Response

**Published:** 2025-03-07

**Authors:** Dominique Riddell

**Affiliations:** Royal Veterinary College, London, UK

Thank you for feedback and for raising these points. We discuss them below and have adjusted our manuscript accordingly.

**Summary.** *I wonder why the authors chose to perform this biomarker study at 12 months, which is quite late in the life of the dogs in terms of their clinical evolution. At an earlier time point, the CK elevation would probably have been more pronounced, and possibly that of other biomarkers, at least the dystromiRs and inflammatory cytokines. Therefore, I think that the conclusion that MYOM3 and CCL2 are not affected by exercise should be drawn with more caution, because it might be different at earlier time points with a more pronounced exercise-induced CK increase.*  

**Response: **Thank you for raising this. We discuss this point specfically below in response to your questions. We have also edited our statements in the abstract and discussion to limit our conclusions to the single timepoint tested in the biomarker-response section of the study.  

**1.** *How is the time not walking measured?*  

**Response: **Time spent not walking (standing, sitting or lying down) was recorded throughout the test (as time continued to elapse on the stopwatch), with total time not walking combined. We have now clarified this in the methods section.  

**2.** *Are the tests filmed and is video analysis used?*  

**Response:** For this work no, they were not filmed. However, we have more recently filmed the 6MWTs with a view, in future to examining gait and/or behavioural differences.  

**3.** *The 6MWT is highly standardized in humans, in particular the instruction to walk as far as possible. Has the test been performed in a walking gait in dogs? It seems difficult to force dogs to maintain a walking gait, and perhaps a bit artificial, so how was this done?*  

**Response: **As you allude to, one of the key challenges when converting the human 6MWT to a canine 6MWT is conveying the instruction to walk as far as possible and achieving cooperation from the dogs. In order to achieve this, the test was standardised as much as possible whilst allowing for encouragement during the test to maintain engagement with the task. Each animal was walked on the handler’s right side, controlled on a leash at the dog’s natural pace around the circuit. Dogs were gently encouraged with voice commands, treats and gentle leash tugs when required but allowed to slow or stop as necessary. We have added detail of our approach to encourage walking at the dog’s natural pace to the methods. All dogs were habituated to the testing area as this was an area they were weighed in twice weekly. As already mentioned in our discussion, 12 week old puppies, regardless of genotype, were easily distracted and this perhaps contributed to the longer time spent not walking in WT dogs at younger ages, whereas more mature dogs walked for a greater percentage of the 6 minutes, suggestive of a greater understanding of what is required of them and their enhanced focus. As the reviewer will no doubt be aware, there are related practical challenges when utilising the 6MWT in young DMD boys and as suggested in the manuscript, direct activity monitoring might help overcome some of the biases or other practical constraints of the 6MWT in dogs.

**4. Do the authors think that cognitive impairments, which they have already described in their model, might affect the performance of DE50-MD dogs in this test?**  

**Response: **This is an important point, thank you for raising it. Yes indeed, the cognitive phenotype could contribute to the performance of the DE50-MD dogs in the 6MWT. In our previous work (Crawford *et al*., 2022), we showed that, while DE50-MD dogs demonstrate comparable rates of learning new tasks to WT dogs, they show reduced focus on the tasks. This may also occur during the 6MWT: time spent not walking could be due to muscle functional deficits or a loss of focus and engagement with the test. DE50-MD dogs also show reduced exploratory behaviour, which perhaps also contributes to a reduced interest in walking around the 6MWT assessment area. It has also recently been shown that DMD patients with neurodevelopment disorders (including autism and attention-deficit/hyperactivity disorder) perform significantly worse in motor function tests than those without such disorders – this was true for a range of timed function tests and the total NSAA score (Keselman *et al*., 2025). We have added discussion of these factors to the manuscript. It will be of interest in the future to evaluate some of these aspects in animals that receive a purely muscle-directed treatment in order to try to separate cognitive from musculoskeletal dysfunction, which is otherwise challenging. Crawford AH, Hildyard JCW, Rushing SAM, et al.: Validation of DE50-MD dogs as a model for the brain phenotype of Duchenne muscular dystrophy. *Dis Model Mech.* 2022;15(3): dmm049291. 35019137 10.1242/dmm.049291 8906169 Keselman D, Glanzman A, Thelen MY, Prosser LA, McGuire J, Matesanz SE. Motor function testing rates and outcomes in Duchenne muscular dystrophy with comorbid autism and attention-deficit/hyperactivity disorder. Neuromuscul Disord. 2025 Jan 30;48:105281. doi: 10.1016/j.nmd.2025.105281. Epub ahead of print. PMID: 39946777.  

**5.** *About the biomarkers.The CK elevation seems mild, but this may be due to the graphical presentation. I would suggest adding a Y-axis cut-off in Figure 5H to make it clearer. It would also be useful to add information about the range of elevation in the results section.*  

**Response: **Thank you for this suggestion: we agree that the graphical presentation compresses the elevation: we selected a log-scale in order to visualise both WT and DE50-MD on the same axis as the results for the 2 genotypes are orders of magnitude apart. Having tried several options for cutting the Y-axis we were unable to improve the clarity significantly. Therefore, we have instead added graphs showing the percentage increase pre- to 3-hours-post 6MWT for the 2 biomarkers that showed a significant increase: these graphs clearly demonstrate the CK and KC-LIKE elevation. We have also added information about the range of elevation in the results section.  

**6.** *I question your decision to perform this analysis at 12 months of age rather than earlier, when the dogs still have myofibers to break down. The CK increase would probably have been more pronounced and the assessment of the sensitivity of the other biomarkers to exercise would have been more reliable.*  

**Response: **We understand the point raised by the reviewer that it would be ideal if we could have examined pre and post exercise blood samples from all dogs at all ages however, a key factor in our decision to conduct the study at this single time point was dog availability and importantly, for ethical reasons (given that this would have very substantially increased the number of times blood was collected from each animal). The study was performed as part of our ongoing natural history work, for which we first established a panel of suitable biomarkers in non-exercised dogs, as well as establishing the utility of the 6MWT itself, before conducting the biomarker response study; however, by this time, all dogs were already recruited and part-way through the study. Hence, we only had sufficient N-numbers of dogs for a well-powered exercise study at 12-months of age. We see the exploration at the 12 month time point as a proof of principle, as our preliminary biomarker work identified the selected markers as highly suitable for differentiating between genotypes in 12-month old dogs. But we agree that it is possible that exercise-induced increases in serum biomarkers with the 6MWT might correlate with the total amount of exercise performed and so it would certainly be interesting to perform this analysis at additional time points, though this would have to be performed in future studies. We have addressed this limitation in the text and have been more cautious with our conclusions in light of our test being restricted to a single timepoint.  

**7.** *In the paper published by Acosta et al. in 2016 on the 6 MWT in GRMD dogs and its effect on CK levels, significant change in CK was detected after the 6 MWT at 6 months of age, but not at 12 months of age. This should be included in the limitations of the study in the Discussion section.*  

**Response: **As discussed, in contrast to the GRMD work, we did find a significant increase in CK at 12-months, indicative of differences between our models, which is corroborated by our other natural history work and is perhaps unsurprising based on the different breeds and sizes. The DE50-MD percentage increase in CK activity (median: 132%; range 59-326) was closer to the 6-month GRMD dogs (median: 104%; range 93-328) than the 12-months GRMD (median: 18%; range -0.4-67.9), despite the 12-month-old DE50-MD and GRMD dogs both walking shorter distances than the 6-month GRMD dogs. Perhaps, had we also performed the biomarker-response study at 6-months, when DE50-MD were capable of walking greater distances, we might have seen a greater CK increase than at 12-months. As above, we agree this is a limitation and have added to the discussion, with reference to the Acosta paper.

# Reviewer report: Reviewer response for version 1

**DOI:** 10.21956/wellcomeopenres.25648.r114903

**Published:** 2024-12-30

**Authors:** Kristy Swiderski

**Affiliations:** 1 The University of Melbourne, Parkville, VIC, Australia

This study outlines a well-designed and well-written evaluation of the 6MWT in healthy and dystrophic dogs, utilizing the DE50-MD model, which has been studied extensively in previous publications from this group. Assessment using the 6MWT every 3 months of age from 3 to 18 months of age provides a comprehensive dataset demonstrating that dystrophic dogs perform significantly worse than WT dogs at all ages. Importantly, the study provides key data demonstrating that some but not all serum biomarkers of dystrophic disease are altered by the 6MWT, and that very little variation was detected between duplicate tests performed 48-72 hours apart, indicating that a single test at each time point would be sufficient in subsequent studies, both of which have relevance to clinical applications.

 As a minor point, analysis of serum biomarkers to determine the impact of the 6MWT was only performed in the 12-month-old dogs, which have undertaken 4 bouts of the 6MWT. Is it likely that the repeated testing may influence these markers? Additionally, given the authors have previously assessed at least some of these biomarkers over these specified ages and demonstrated that some are not increased in younger dogs, is it possible that the 6MWT may have greater influence on these markers if tested in younger dogs? Acknowledgement of these points in the discussion would be useful.

Is the work clearly and accurately presented and does it cite the current literature?

Yes

If applicable, is the statistical analysis and its interpretation appropriate?

Yes

Are all the source data underlying the results available to ensure full reproducibility?

Yes

Is the study design appropriate and is the work technically sound?

Yes

Are the conclusions drawn adequately supported by the results?

Yes

Are sufficient details of methods and analysis provided to allow replication by others?

Yes

Reviewer Expertise:

skeletal muscle physiology, skeletal muscle function, myogenesis, signaling

I confirm that I have read this submission and believe that I have an appropriate level of expertise to confirm that it is of an acceptable scientific standard.

# Response

**Published:** 2025-03-07

**Authors:** Dominique Riddell

**Affiliations:** Royal Veterinary College, London, UK

Thank you very much for your supportive feedback. We are happy to address your questions.

**1.** *As a minor point, analysis of serum biomarkers to determine the impact of the 6MWT was only performed in the 12-month-old dogs, which have undertaken 4 bouts of the 6MWT. Is it likely that the repeated testing may influence these markers?*

**Response: **Regarding the impact of 4 bouts of 6MWTs on the serum biomarkers: the distance walked by the dogs in the 6MWT is relatively short, with a percentage of the test spent resting, and the test is conducted infrequently, at 3-monthly intervals in animals that between tests have access to outside runs and grass paddocks when they can exercise freely. Thus, we deem it unlikely that the 6 minutes of mild, intermittent exercise would have an effect that lasts until the following test conducted 3-months later. However, it is possible that having performed the test 4 times, the dogs may remember previous tests and therefore better understand what is expected of them. In WT dogs, there is a reduced time spent not walking as dogs age, which we have speculated is due to learning what the test involves and/or increased focus as the dogs mature. This effect is not seen in the DE50-MD dogs however, who actually spend more time not walking during the 6MWT as they age – likely due to reduced functional capacity of their muscle. Thus, we think it unlikely that repeated bouts of testing will influence analysis of serum biomarkers in 12-month-old DE50-MD dogs.

**2.** *Additionally, given the authors have previously assessed at least some of these biomarkers over these specified ages and demonstrated that some are not increased in younger dogs, is it possible that the 6MWT may have greater influence on these markers if tested in younger dogs? Acknowledgement of these points in the discussion would be useful.*

**Response: ** Of the biomarkers measured in the study, we have previously shown that serum CCL2, MYOM3, MSTN, CK activity and miR-1, -133a and miR-206 levels are all significantly different in DE50-MD compared to WT at all timepoints (3, 6, 9, 12, 15 and 18-months). However, there are age-related differences: CCL2, MYOM3 and CK activity all peak in serum at 6-months of age and decline steadily thereafter, while MSTN shows the greatest difference between genotypes between 9-12 months, and the 3 miRs show no significant variation with age. In comparison, the rest of the inflammatory cytokines show greater variation between ages and within genotypes, with significant differences typically seen at 9-months of age for the interleukins, while mean KC-LIKE is elevated at 3, 12, 15 and 18-months of age. We have speculated that the 6-month peak and subsequent decline in MYOM3 and CK activity is a result of a decline in physical activity conducted day-to-day by the dystrophic dogs, as a result of disease progression. In support of this, beyond 6-months, DE50-MD dogs have reduced capacity to withstand eccentric contraction (Riddell *et *al., 2023) and, as we have shown in this study, walk significantly shorter 6MWDs. As 6-month-old DE50-MD dogs walk greater absolute and stride-length adjusted distances at 6- compared to 12-months, it is possible that the greater the distance walked the greater the increase in serum markers of muscle damage. Thank you for your suggestions: we have added discussion of the potential influence of age on the exercise-biomarker response test into our manuscript.
